# “I know I shouldn't but …” the inevitable tension of using workarounds to be a “good nurse”

**DOI:** 10.3389/frhs.2025.1579265

**Published:** 2025-07-29

**Authors:** Deborah Debono, David Greenfield, Wendy Lipworth, David J. Carter, Deborah Black, Reece Hinchcliff, Jane Ellen Carland, Jeffrey Braithwaite

**Affiliations:** ^1^ School of Public Health, Faculty of Health, University of Technology Sydney, Sydney, NSW, Australia; ^2^ Faculty of Medicine and Health, University of New South Wales, Kensington, NSW, Australia; ^3^ School of Humanities, Macquarie University, Sydney, NSW, Australia; ^4^ Faculty of Law and Justice, University of New South Wales, Kensington, NSW, Australia; ^5^ The Faculty of Medicine and Health, The University of Sydney, Sydney, NSW, Australia; ^6^ Department of Management, Griffith Business School, Griffith University, Brisbane, QLD, Australia; ^7^ School of Public Health and Social Work, Faculty of Health, Queensland University of Technology, Brisbane, QLD, Australia; ^8^ Department of Clinical Pharmacology and Toxicology, St Vincent's Hospital Sydney, Darlinghurst, NSW, Australia; ^9^ School of Clinical Medicine, St Vincent's Healthcare Clinical Campus, Faculty of Medicine and Health, University of New South Wales Sydney, Darlinghurst, NSW, Australia; ^10^ Australian Institute of Health Innovation, Macquarie University, Sydney, NSW, Australia

**Keywords:** workaround, electronic medication systems, medication, nurse, patient safety

## Abstract

**Introduction:**

Rules, policies, and technologies are increasingly introduced in healthcare to reduce complexity and iatrogenic harm. One example is the implementation of Electronic Medication Management Systems (EMMS) to minimise medication errors. However, in hospitals where nurses primarily administer medications, research shows that nurses often adopt “workarounds” to overcome barriers in medication administration. This study explored how nurses experienced and perceived the use of workarounds in their daily medication administration practices. Understanding these feelings is crucial, as they are linked to both patient safety and staff retention.

**Methods:**

This ethnographic study was conducted in six wards in two Australian hospitals across 91 shifts, 46 interviews, seven focus groups, and member-checking sessions with nurses and EMMS stakeholders (*N* = 113 participants). Data analysis used a general inductive approach.

**Results:**

Nurses described positive, negative, ambivalent, and conflicting feelings about using workarounds. Some denied the use or tolerance of workarounds, despite them being routinely observed. Most reported a tension between the perceived necessity of workarounds, reluctance to deviate from policy, and the desire to be a good nurse. Workarounds were seen both as the trademark of an expert, mindful nurse and as deviations from the rules, unsafe for both patients and nurses.

**Discussion:**

This study demonstrates challenges to patient safety associated with the tension between the necessity of workarounds and the desire to adhere to policy. This can create stress and anxiety among nurses. They experience a tension at the intersection of the necessity of workarounds to deliver care, to be a good nurse, and the desire to adhere to policy. The associated stress and anxiety can lead to burnout, professional disengagement, and attrition. The study proposes solutions to manage challenges associated with workarounds.

**Conclusion:**

Workarounds are an inevitable aspect of healthcare delivery in response to standardisation. Negative perceptions of workarounds may inadvertently contribute to the very harm that standardisation seeks to prevent. A more open dialogue about their use is essential. Recognising their inevitability and equipping nurses to manage them constructively is key to reducing stress, preventing burnout, and enhancing patient safety.

## Introduction

The development of rules, policies and technologies based on how care is thought to be delivered—work-as-imagined—without input from those delivering the care—work-as-done, leads to workarounds (1). Increasing numbers of workarounds to rules, policies and technologies introduced to reduce complexity, ironically increase it (2). Increased complexity has been linked with iatrogenic harm (3) which affects one in ten patients, accounts for over three million deaths per year (4) and is the tenth leading annual cause of death globally (5). It has been estimated that 50% of this harm is preventable and that medication-related incidents account for half of the harm (4). To mitigate iatrogenic harm, healthcare has adopted safety strategies from high-risk industries such as aviation and motor racing (6, 7–10). These include the implementation of technology, team-based training, and the development of rules, policies, and guidelines aimed at enhancing patient safety (11–13). A key strategy involves the organisation and standardisation of clinical practices (11, 14, 15), which when undertaken without input from those delivering the care, can introduce new risks, such as familiarity that leads to decreased vigilance or over-reliance on a single type of technology (16), and lead to workarounds.

The inherent complexity and unpredictability of healthcare often require practitioners to respond swiftly and adaptively. When resources are limited, this may necessitate creative problem-solving that occasionally involves deviating from established protocols. As highlighted during the COVID-19 pandemic, nurses need to continuously reassess, reprioritise, and adapt to shifting circumstances, adapting to the complexity and unpredictability of care delivery in environments that frequently include operational failures and uncertainty (17, 18). While adherence to standardised procedures is said to underpin safe practice, there are instances where delivering safe care to an individual patient may require clinicians to work around certain policies or use technologies in unintended ways. Thus, workarounds can represent acts of resilience and serve as valuable opportunities for learning and system improvement (1).

While workarounds—also referred to as shortcuts, deviations, or temporary fixes—are common across everyday life and professional settings, including healthcare, engineering, and IT, published reviews on workarounds and the related concept of safety violations highlight a lack of clear definitions (19–24). This study adopts the definition of workarounds as practices that diverge from organisationally prescribed or intended procedures to circumvent actual or perceived barriers to achieving a goal, or to achieve them more readily.

Workarounds are widespread in healthcare (25, 26–29), where clinicians are often described as “masters at workarounds” (30:52). Their use has been documented across various contexts, including electronic health records (EHR) (8, 28, 31–34), high-pressure workloads (22, 35, 36), system inefficiencies (37, 38) and electronic medication management systems (EMMS) (19, 20, 38, 39). For example, in barcode medication administration (BCMA), nurses have been observed bypassing the requirement to scan patient wristbands by instead scanning barcodes on stickers or paper (40). Despite their ubiquity, workarounds almost never appear in official documentation such as policies and procedures and are rarely discussed openly and often omitted from formal accounts of nurse behaviour, making their contribution to care delivery and outcomes difficult to capture.

While traditional safety approaches (Safety-I) often view deviations from policy as threats to safety due to their potential to introduce variability and error (37, 41–43), more recent literature (Safety-II paradigm) offers a more nuanced view. Safety-II emphasises understanding of how people adapt successfully in complex, variable environments, suggesting that not all deviations are harmful, and some are in fact necessary for safe care (44). In this view, safety emerges not solely from compliance, but from the capacity of systems and workers to adapt. Nonetheless, organisational responses to safety tend to align more with Safety-I assumptions, where policy deviations are seen as inherently risky and in the minds of some, unacceptable, unethical (45, 46) and at times illegal (47). This may reflect regulatory pressures or risk management norms, rather than empirical consensus. By contrast workarounds reflect the real world of nursing work, in which they are often seen as essential to embody the traits of a “good nurse” such as time efficiency, a focus on patient safety, patient-centred care, and teamwork (1, 48–50). These competing perspectives, may create a source of stress when nurses feel compelled to use workarounds, potentially compromising nurses' well-being and thereby undermining patient safety (43, 51).

This is a critical issue, particularly as stress and burnout are correlated with patient safety and staff attrition (43, 51). Furthermore, the link between burnout and workarounds has been made clear in other work (43, 52, 53). Where autonomy is low and emotional stress or exhaustion is high, self- and supervisor-reported workarounds become more prevalent. Indeed, burnout can lead to more workarounds (53–55), lowered professional engagement (54, 55) and more adverse events (56, 57). The cyclical nature of burnout, lowered nurse engagement and associated workarounds can pose significant safety risks to patients and decrease work satisfaction for nurses, contributing to intent to leave and turnover rates (43, 55, 58).

The ability to independently resolve issues through workarounds may be interpreted as a demonstration of professional competence. Given their ubiquity and paradoxical role—both enabling care and carrying negative connotations—it is important to understand how and why nurses use workarounds. While there is growing interest in the types of workarounds employed and the motivations behind them (19, 28, 29, 39, 48, 59), less attention has been paid to how nurses feel when engaging in such practices. While noting the ubiquity of different types of workarounds across different contexts, given the use of workarounds occurs most frequently when implementing new technology (46%) and when administering medications (31%) (22), this study sought to address this knowledge gap by examining how nurses feel about using workarounds when administering medication using EMMS in everyday practice.

Despite variable evidence supporting their effectiveness (60–62), EMMS have been introduced in high-income health systems, primarily to: provide information support; and promote standard practice to reduce the incidence of medication errors (37, 63). Within an EMMS, electronic medication administration records (eMARs) provide a standardised process and record of a patient's ordered and administered medications. EMMS and eMARs—through the physical items and computerised programs that comprise them, and the policies that direct their use—dictate nurses' actions and their timing, establishing an order and routine to medication practice; thereby aiming to standardise practice, promote safety and the minimisation, or in an ideal world, elimination of errors. Mobile computer workstations, workstations on wheels [WOWs, known as computer on wheels (COWs) at the time of data collection], enable EMMS and eMARs to be taken to the patient's bedside.

Nurses' use of workarounds during medication administration using EMMS provides a lens through which to explore nurses' experiences and emotional responses when using workarounds. The results of this study can inform the development of strategies, including both practice and policy, to enhance patient safety.

## Methods

We purpose-designed a multi-methods ethnographic study to examine the workaround theory–practice gap and reveal the intricacies of complex nursing work environments; the study involved process mapping, observation, focus groups and interviews. Workarounds, a form of articulation work (practices that get work back on track) (64), often remain invisible in formal accounts of nursing practice because these non-sanctioned strategies may involve deviating from official policy. Nurses may interpret their actions differently from others and, being accustomed to resolving problems independently, may not recognise their solutions as workarounds or deem them noteworthy. Given their informal and context-specific nature, observing workarounds in practice was essential. Ethnography was identified as the most appropriate methodological approach, as it prioritises first-hand engagement with the setting under study and situates observed behaviours within their broader organisational and cultural context.

Participants included EMMS implementation stakeholders and nurses from six clinical units across two hospitals, each employing distinct EMMS platforms. A triangulated data collection strategy was employed, incorporating direct observation, individual interviews, and focus group discussions. Data were collected across all shifts and days of the week to maximise variation and reduce the likelihood of overlooking key phenomena influencing nurses' enactment, rationale, and experience of workarounds. Data were analysed using a general inductive approach, which facilitated the emergence of themes grounded in the data and aligned with the study's research questions.

To establish a clear understanding of the intended workflow for medication administration, a “gold standard” process map was developed for each participating hospital. These visual representations outlined the prescribed steps for administering medications using the EMMS. The maps were informed by medication policy document analysis, participation in eMAR training sessions and consultation with EMMS implementation experts at each hospital (65). The process map at one hospital was structured around route of administration, and by regulatory requirements (medications requiring a witness, a co-signer, or neither) at the second hospital. The process maps were instrumental in identifying where workarounds occurred within the medication administration process.

### Setting

Data were collected at two metropolitan university-affiliated teaching hospitals in Australia. Each hospital (Hospital A and Hospital B) had over 300 beds and well-established EMMS, with roll out commencing six years prior to the study. Three units were sampled at each hospital and comprised surgical, haematology, and four medical units [three 34-bed units (A1, A2, and A3) and three units with either 26 or 28 beds (B1, B2, and B3)]. Different models of nursing care (patient allocation or team nursing) and EMMS were used at each hospital.

### Sampling strategy

The study employed a non-probabilistic, purposive sampling strategy to capture a specific group with experience and knowledge of EMMS. Novice and experienced nurses who used EMMS and EMMS implementation stakeholders (staff involved in implementation and support of EMMS) were included in the sample. To accommodate ever-changing demands across shifts nurses were invited by the researcher to participate if they were available when data collection was conducted. One nurse declined to participate. Participants were allocated a unique identification number.

### Recruitment

Information sessions were conducted with the EMMS stakeholders and on the participating units where nurses were invited to join the study. Participant information sheets and consent forms were provided. A researcher visited the units several times prior to commencement of data collection to invite participants to the study, provide information as needed and orientate to the research task.

### Participants

A convenience sampling approach was used and nurses working on the study units (*N* = 6) in two hospitals were invited to participate in the study if they were available during the data collection period. There were 113 participants in total, comprising 60 nurses (A1 = 26, A2 = 20, A3 = 14) and four EMMS implementation stakeholders at Hospital A, and 46 nurses (B1 = 15, B2 = 17, B3 = 14) and three EMMS implementation stakeholders at Hospital B. Most participants were full-time registered nurses with more than one year of experience.

### Data collection methods

**Observations:** A combination of non-shadow and shadow observation was employed. *Non-shadow observations* collected data to build a picture of normative operating behaviours, assumptions, attitudes, interactions and beliefs. Rather than closely observing medication administration, non-shadow observations noted practices, communication, artefacts (EMMS, notes and equipment), rituals and symbolic behaviour visible on the unit. Clarification of observed behaviours and interactions was sought from participants through opportunistic questioning—when prompted by participants, during shifts without disrupting workflow, at shift end, during interviews, or at the next suitable encounter. This process supported the interpretation of how nurses made sense of their actions and contributed to a contextualised account of each setting, including events and behavioural rationales from the participants' perspectives.

*Shadow observations* involved observations of a nurse during medication administration using the process map to identify workarounds. They were also conducted during other activities that did not involve patient personal care. Shadow observations were conducted to examine how nurses administered medications and engaged in related tasks, with a particular focus on identifying workarounds, their contextual influences, and how nurses responded to their own and others' use of such practices. Variations from the standard process were explored with participants to determine whether they constituted workarounds. Observations were conducted during morning (*n* = 44), evening (*n* = 35) and night shifts (*n* = 12) seven days a week. Given the temporal influence, and inseparability, of medication work from nurses' other work (66), nurses' use of workarounds with EMMS was examined in the context of nurses' work across a shift and, as far as possible, for complete shifts. Observation times ranged between two-and-a-half to nine hours twenty minutes. All activities observed during the time frame were documented, including omissions of expected behaviour. Spradley's framework (67) of generalised concerns (Table 1) guided note-taking to structure data recording. Additionally field notes captured nurses' explanations and reflections on their rationale for and experiences of using workarounds. These discussions during observations supported ongoing formative member checking.

**Table 1:** Strategies for ethnographic observation note taking (67:78).

Item	Focus
Space	The physical place or places
Actor	The people involved
Activity	A set of related acts people do
Object	The physical things that are present
Act	Single actions that people do
Event	A set of related activities that people carry out
Time	The sequencing that takes place over time
Goal	The things people are trying to accomplish
Feeling	The emotions felt and expressed

**Interviews and Focus Groups:** Face-to-face semi-structured interview and focus group data complemented observational data. Semi-structured interviews were conducted to garner individual's perspectives about workarounds. Focus groups were employed to generate interaction to capture the collective view on workarounds. Development of the interview and focus group topic guides was informed by literature and the expertise of the research team. Open-ended questions explored participants' experiences of using EMMS and workarounds (Tables 2, 3).

**Table 2:** Semi-structured interview topic guide.

Topic question
Could you explain the electronic medication management system that is used in this unit to me please?
Can you tell me about the medication process that is used in this unit? [Prompts: Is there a medication round, nurse dedicated to medication delivery, pharmacy round etc.]?
Can you tell me about how has using the electronic medication management system changed aspects of your work?
Are there times when it is difficult to use the electronic system in administering medication? Can you tell me about some of the things that make it difficult?
Can you tell me about what do you do when something makes it difficult to get the medication to the patient?
Does everyone use the same practices to get the medication to the patient? Can you tell me about how the practices differ between nurses?
Can you tell me about whether and how you workaround the system to get the medication to the patient?
Can you tell me about whether and how other people workaround the system to get the medication to the patient?
Would you explain for me if there are times when it is OK to work around the system to get the medication to the patient and when it is not OK? Is this the same for everyone?
Can you tell me about times when it is OK for some nurses to work around the system the system to get the medication to the patient but not OK for others to work around?
Are there times when it is easier to use the electronic system in administering medication? Can you tell me about some of the things that make using the electronic medication management system easier?
Can you tell me what impact you think the electronic medication management system has had on quality and safety?
What sort of things impact on the use of the electronic medication management system? [For example, experience with the system, business of the shift, staff levels]

**Table 3:** Focus group topic guide.

Topic question
Can you tell me about your experiences using electronic medication management systems?
Are there any characteristics of the organisation, unit environment or set up that make it less or more difficult to use the electronic medication management system?
Can you tell me about any characteristics of the electronic medication management system that make it easy or difficult to use?
Can you tell me about any patient characteristics that make it harder or easier to use the electronic medication management system?
Can you tell me about circumstances when it is more difficult to use the electronic medication management system? (Prompts: Busy, short staffed, patient factors e.g., patient sleeping)
Can you tell me about any positive and negative unintended consequences of electronic medication management systems?
I am really interested in whether everyone uses the same practices to get the medication to the patient. Can you tell me about how the practices differ between nurses?
Can you tell me about whether and how you workaround the system the system to get the medication to the patient?
Can you tell me about whether and how other people workaround the system the system to get the medication to the patient?
Would you explain for me if there are times when it is OK to work around the system to get the medication to the patient and when it is not OK? Is this the same for everyone?
Is there anything else you would like to add?

Interviews and focus groups were conducted in participants' work units or offices and transcribed for analysis. Interviews lasted between 15 and 89 min (mean: 34 min; median: 31 min). Focus groups with nurses ranged from 37–51 min (mean: 42 min; median: 41 min), while those with EMMS implementation stakeholders averaged 97 min. A summary of data sources can be found in Table 4.

**Table 4:** Summary of data sources.

Data source	Activities
Observations	91 shifts or part thereof (30 non-shadow; 61 shadow)
Interviews	46 participants (45 nurses; 1 EMMS implementation stakeholder)
Focus groups	7 focus groups [5 with nurses (*n* = 5; 4; 6; 3; and 6) and 2 with EMMS implementation stakeholders (*n* = 4; and 2)]
Member checking sessions	3 (2 group and 1 individual)

### Data analysis

Data analysis employed the general inductive approach (68). In keeping with a general inductive approach to analysis a blended “grounded” coding for themes and concepts, and “responsive formal coding,” which focused on coding against the research question rather than line-by-line coding was employed (69). The observational field notes and focus group data were coded for categories identified in the interviews. QSR NVivo 10 software was used to manage interview data, and an Excel spreadsheet used for the field notes and focus group material.

#### Step 1: familiarisation with the data

The initial phase involved an immersive reading of interview transcripts and field notes, during which preliminary patterns, reflections, and ideas were noted. These were informed by contemporaneous reflections recorded during data collection. Transcripts were imported into QSR NVivo 10 software to facilitate systematic data management throughout the analysis.

#### Step 2: coding framework development

##### Identifying workarounds

Given the study's focus on nurses' use of workarounds, data were examined for behaviours aligning with the definition of workarounds. Process maps served as a benchmark against which observed or described behaviours were compared. Deviations from the prescribed process, such as omitting a formal identification check or using informal identifiers (e.g., bed numbers or handwritten notes), were coded as workarounds when they were used to overcome perceived time or workflow barriers.

##### Developing additional codes

Following the identification of workarounds, relevant transcript sections were re-examined to generate descriptive, content-driven codes. Coding was iterative and reflexive, with new codes prompting re-evaluation of previously coded data. Multiple codes were often applied to the same data segments. Rigorous attention was paid to remain grounded in the data, avoiding over-interpretation. Coding decisions were discussed with research team members and a qualitative coding expert.

#### Step 3: thematic refinement and conceptual development

Coded data were grouped into themes representing similar phenomena related to workarounds. Themes were then examined for interrelationships, forming broader categories and, ultimately, abstract concepts. Subsequently, observational field notes and focus group data were coded using the interview-derived categories. This process aimed to enrich and interrogate the emerging concepts while remaining open to new categories. An Excel spreadsheet was used to track category occurrences, with an additional column for novel insights not captured by existing codes.

### Ethics

Ethics approval was granted by a Lead Human Research Ethics Committee (HREC), (HREC/10/XXX/116) (HREC name deidentified with XXX) and ratified by University of New South Wales HREC (HC09223). Participants provided written consent which was reestablished throughout the data collection process.

## Results

Observational data revealed both similarities and differences in local practices, communication preferences, workflow, and nursing care models across the study units. The physical layout of units limited visibility across sections, potentially affecting staff awareness of colleagues' workloads. In all units, oral medications were dispensed by pharmacy and stored in locked bedside drawers, while controlled, refrigerated, and injectable medications were stored in the medication room. A locked metal cupboard (DD cupboard) housed Schedule 8 and Schedule 4D medications (DD medications), with corresponding burgundy A4 drug register books nearby. Administering DD medications required two nurses to verify the order, reconcile stock, witness preparation and administration, dispose of any residue appropriately, and sign the register and medication chart. These procedures were governed by legislation aligned with nurses' scopes of practice.

Medication rounds were structured, the heaviest workload occurring in the morning following handover, when demand for WOWs was greatest. Each unit had between five and ten WOWs available. Two models of nursing care were observed across the participating units: a patient allocation model at Hospital A and a team-based model at Hospital B. At Hospital A, individual nurses were assigned specific patients and held responsibility for their care throughout the shift. In contrast, Hospital B employed a team nursing model, where a group of nurses, led by a team leader, collectively cared for a group of patients. Staffing strategies also differed between the hospitals. At Hospital A, workforce shortages were primarily addressed through the use of overtime, agency, and casual pool nurses. At Hospital B, staff were more commonly redeployed from other wards to cover shortages, with minimal reliance on agency or casual staff during the study period. In addition to using different EMMS platforms, the hospitals differed in their EMMS access protocols—one granted eMAR access to all medication-endorsed nurses post-training, while the other restricted access to permanent staff who had completed training—and in their nursing care models (team-based vs. patient allocation). The eMAR is accessed at stationary desktop computers or via laptops mounted on trolleys, “workstations on wheels” (WOWs).

The study also identified several key differences in the configuration and functionality of the eMAR systems across the two participating hospitals that were relevant to nurses' use of and perceptions of workarounds when administering medication (Table 5). For example, during initial observations at Hospital A, it became apparent that the overdue medication alert (OMA) within the EMMS elicited strong emotional reactions from nursing staff. Nurses at Hospital A expressed more pronounced emotional responses to OMAs than their counterparts at Hospital B. At Hospital A, OMAs were prominently displayed next to patient names in the EMMS interface. Patient allocations were recorded in a staffing book and, in some units, also displayed on whiteboards in the main office—commonly referred to as the “flight deck”—which served as a central hub for clinical staff. This visibility made it easy to identify which nurse was responsible for any overdue medications. Nurses at Hospital A reacted to OMAs as indicators of personal failure, describing feelings of stress, frustration, worry, and inadequacy. Although formal reprimands were rare, the alerts created a sense of time pressure and concern about professional judgement. Nurses associated the alerts with being seen as “not coping”, “lazy”, or “neglecting patient care”.

**Table 5:** Differences between site-specific electronic medication administration record (eMAR) features identified as relevant to this study.

Feature	Hospital A	Hospital B
Access to eMAR	All nurses endorsed to administer medication, including agency and casual staff, following training at the commencement of a shift	Only permanent staff and selected casual staff who regularly worked on wards using EMMS after completing training sessions
Concurrent access by multiple users to the same eMAR	No—when one user opened the eMAR, it was blocked to other users	Yes—several users were able to log in and be active in the same eMAR at the same time
Blocks at administration based on scope of practice	Users were blocked from confirming medication administration in the eMAR for medications they were not endorsed to administer. For example, Endorsed Enrolled Nurses (EEN) were able to log in to the eMAR but not confirm the medication administration of subcutaneous Heparin	No blocks
Automatic log out time	Shorter than ten minutes	Longer than ten minutes
Required fields for checking nurses to complete when checking medication (co-signing or “checking” which is different from medication administration requiring witnessing)	The nurse witnessing the administration of injections (intravenous, subcutaneous, intramuscular) needed to enter a username and password for the administration to be recorded as completed in the eMAR	The administering nurse typed the name of the checking nurse in the Comment Box—the checking nurse was not required to enter any information
Pharmacist instructions	At Hospital A a purple triangle indicated pharmacist instructions that were required to be read. It was necessary that nurses confirm that the pharmacist’s instructions had been accessed and read in order to proceed with the medication administration	It was not necessary to confirm that pharmacist instructions had been accessed and read to proceed
Additional required fields to proceed to record administration	A reason needed to be entered when withholding a medication	A reason needed to be entered when withholding a medication.
		The pulse rate for Digoxin and blood glucose level (BGL) for insulin needed to be recorded for the administration of these medications to be completed in the eMAR
Functionality to manage intravenous therapy	No	Yes
Insulin	Paper-based medication charts also existed for insulin. The EMMS alerted the nurses that there was an insulin order on a paper chart, “Insulin Paper Chart exists for this patient”, so nurses need to mark that the insulin has been administered on the electronic chart and on the paper chart	Insulin was ordered in the eMAR; there was no paper chart for insulin in the wards that used EMMS
Visibility of overdue medication alert (OMA)	Visible next to the names of patients with medications that were overdue at the step of the process when the ward was selected in the eMAR	Not visible until the individual patient’s eMAR was opened

(48:p.100).

96: I was beside myself when I saw that clock. I'd failed. When I first came−‘No, No’−I thought. I was really, really stressed because I wasn't getting through the medication by nine o'clock but that was because I saw the clock. (Focus Group: Nurses_ID_4)

At Hospital B, the OMA was only visible when a clinician opened an individual patient's electronic medication chart, making it less prominent than at Hospital A. This reduced visibility was coupled with a team-based model of nursing care, in which responsibility for medication administration was shared among team members. Participants at Hospital B generally viewed the OMA as a simple reminder rather than a source of pressure.

I like it because it's readable. It's going red when it's overdue or you just forgot about it, or if we don't have any stock in the ward, it goes red. It means that you didn't give it−gives the nurses a warning. (Interview: Nurse_42)

To avoid being late with medications, in both hospitals nurses were observed to use a variety of workarounds. For example, the nurse:

put the meds in the med cup and put another cup on top of it−put on something, like a piece of paper or Alco wipe, what the bed number is… line the med cups up and then take them to the patients one after another [Field Notes Observation (FNO): _111_AM]

### Observed workarounds

Workarounds were employed in the use of EMMS for medication administration. These workarounds were applied either individually or in combination with others, initially giving the impression of considerable variability. However, discernible patterns emerged in how these combinations were enacted, explained, and experienced by nurses. The variation observed in the workarounds can be understood as resulting from the combination of each process step workaround with none, one, or multiple others. The workarounds presented in Table 6 are illustrative rather than exhaustive examples of observed practices.

**Table 6:** Examples of workarounds.

The way EMMS was intended to be used	Observed workarounds
Responsive electronic medication Administration record (eMAR) on the Workstation on Wheels (WOWs)) taken to the bedside to enable the 5/6Rs when administering medication	The eMAR was accessed via the WOW left in the corridor or on the desktop computer when preparing, checking and administering medication
	The bedside medication drawer was taken to the WOW shelf when the WOW was parked in the corridor
	The medications for administration were memorised without eMAR at bedside
	Medications were administered from a printed record of the eMAR and signed off in the eMAR before or after administration
	Workarounds used to confirm patient identification: patient identification details were memorised, physical markers such as bed numbers or patient names written on Alcohol swabs or the bottom of kidney dishes additive labels or pieces of paper put in the medication cups or kidney dishes, the patient sticker in the bedside folder used to cross check the information on the patient’s identification band, familiarity with the patient and the patient response to being addressed by name was used as a form of identification check
	Patient allergy information was memorised
	The medications for administration were memorised without eMAR at bedside
	Medications were administered using a printout of the eMAR
eMAR signed off at time of administration	The medication was signed off prior to administration
	The medication was administered prior to it being ‘available for administration’ in the eMAR and signed off later when it became available in the eMAR
	Informal communication with colleagues about medication administration
	Medication administration history checked in the eMAR at the point of medication administration at bedside
	Medication was administered before it became ‘available for administration’ in the EMMS and signed off in the EMMS when it later became ‘available for administration’
	Doctors entered STAT medication orders when the nurse could not override the prescribed time for a daily medication
	Informal communication with colleagues about medication administration
The overdue medication alert used to highlight when a medication was late	Medications were ‘delayed’ in the eMAR to remove the visible overdue medication alert (OMA) if they were more than one hour overdue
Each patient’s medications are prepared and administered individually	Medications prepared for more than one patient at a time (‘batching’)
	Physical markers such as bed numbers or patient names written on Alco wipes, the bottom of kidney dishes, additive labels or pieces of paper put in the medication cups or kidney dishes
	Second medication cup was put on top of first medication cup to minimise the risk that the medications would fall and become mixed up with those of another patient
Two nurses required to check the 5/6Rs at the bedside together and witness administration of certain medications and eMAR signed at the time of administration	The nurse who administered the medication was not accompanied to the bedside and medication was signed as administered by both the checking and administering nurse at the preparation and checking step of the medication administration process
‘Checking’ nurse required to check the medication against the eMAR	The medication was checked without the eMAR by relying on familiarity with the patient or their trust of the administering nurse
The administering nurse should be logged into the eMAR	The nurse recorded as the checking or witnessing nurse in the eMAR administered the medication
Some medications could be nurse-initiated using the EMMS when required	Doctors entered STAT medication orders in the eMAR when the nurse did not know how to nurse-initiate the medication in the eMAR
Medications should only be administered from unexpired prescription orders	Medications administered from an active electronic order that was believed to have legally expired
Single patient eMAR open at a time	Nurses opened multiple eMARS at a time (Hospital B)
Medications administered should be dispensed by the hospital pharmacy and stored in patient’s locked bedside medication drawer (Not applicable for DD medications, medications for injection and medications requiring refrigeration)	Nurses ‘borrowed’ medication from the patient’s home stock or from other patients or units
Medications only stored in medication room or locked bedside drawers	Medications stored on the shelves of the WOWs, nurses’ station drawers and nurses’ pockets
Medications administered only by nurses endorsed to do so	Medications were given to Assistant In Nursing (AINs) or relatives, or left on the bedside locker

(48:pp.127–130).

### How nurses felt about using workarounds

#### Overarching duality of feelings

Nurses had mixed feelings about using workarounds. Some felt uneasy or worried about possible consequences, while others saw them as a sign of being resourceful and resilient. How individual nurses felt about using workarounds was influenced by seniority and experience, ward culture, type of task and workaround required. Across both hospitals, participants identified specific units where deviating from EMMS policy through workarounds was not considered acceptable and therefore using them evoked negative emotions. Casual pool nurses, who worked across multiple units, described how local norms could either discourage or enable such practices. While some participants admired units that strictly adhered to policy, others viewed them less favourably—citing, for example, that nurses on a particular ward often worked through breaks and stayed late due to their avoidance of workarounds. Whether tasks were left for the next shift was influenced by unit culture. In some units, nurses expressed concern about being perceived as lazy if medications were delayed or tasks left incomplete. In these settings, workarounds were more commonly used to maintain an appearance of efficiency. Participants also reported adopting local workarounds when working on unfamiliar units to avoid disrupting team routines.

One of the pool nurses explains that when you work on different wards you play by their rules. Some wards have a slightly different way of doing things, different preferences—the nurses get used to how it is done on the ward. [Documented conversation as part of Field Notes Observation (DC/FNO): _57_PM]

#### Negative emotions

Feelings of uncertainty and concern about professional retribution surrounded workarounds. Nurses reported feeling unsettled at their use, particularly as the official stance on workarounds was that they posed a risk to patient safety and compromised staff integrity. They also described tension, powerlessness and fear when management put them in a position in which they were expected to not follow policy. The perception was that in some instances “management” tacitly expected nurses to break policy and systematically “turned a blind eye” to this. At the same time, nurses felt that were an incident to occur following a workaround, the organisation would not support them because they had not followed policy. Nurses frequently suggested that punishment or reprimands were more likely for some workarounds than others. For example, from the field notes during observation of several nurses having a discussion at a nurses' station between a casual RN and permanent junior RNs:

The nurses are discussing the organisational policy, which is that permanent staff need to hold ‘the keys’—not casual and not agency. However, they said, they will send a casual to a ward and if necessary the casual will be In-charge—seniority over permanency when it suits the organisation—one of the casual nurses says, that the management turns a blind eye, and asks—‘What are the casual staff supposed to do? But if there is an incident**, I am hung out to dry** because I know that it was against hospital policy for a casual to hold the keys. [Documented conversation as part of Field Notes Observation (DC/FNO): _126_PM]

Nurses indicated that there were some policies that could be worked around and others that could not; for instance, if a patient had severe chest pain in the presence of the doctor, nurses could administer intravenous morphine, but were not permitted to do so at any other time. Similarly, when medication orders had expired but they could not get a doctor on the unit, nurses described feeling conflicted between administering the medication from an “expired” order for the patient's benefit and not doing so to protect themselves from professional recriminations. In such instances, failing to use a workaround to administer medication was considered potentially harmful to the patient but, at the same time, doing so was perceived as having possible professional ramifications.

Other examples of using workarounds to support patient safety included numerous observations of nurses not taking the WOW to the bedside when the patient was infectious or isolated for immunosuppressed:

A nurse justifies why she left the COW at the door, explaining that you have to weigh up the risks—risking spread of MRSA [Methicillin-Resistant Staphylococcus Aureus] and VRE [Vancomycin Resistant Enterococcus] vs. not following the 5Rs* of giving medication. [(DC/FNO): 204_Morning Shift]

* The 5Rs of medication safety: right patient, right medication, right dose, right route, right time

Participants acknowledged that while certain workarounds reduced the risk of cross-infection, they simultaneously heightened the risk of medication errors. To mitigate this, nurses employed a range of secondary workarounds to verify medications and patient identities against the eMAR. These included writing medication names on paper, preparing ward stock medications in the medication room, labelling medication cups with patient details, and memorising information from the eMAR. Some printed the eMAR and used it within isolation rooms, discarding it there to prevent contamination, and later signed off medications outside the room. Others avoided bringing the COW to the bedside by transporting the medication drawer to the COW shelf, which they cleaned afterwards. In certain cases, a colleague would stand at the door and read out eMAR details while the nurse prepared and administered medications, particularly for controlled drugs.

Many nurses were reluctant to discuss workarounds. Their body language, hushed tones, and descriptors such as “***dodgy***” and “***naughty*”** conveyed their discomfort. Phrases such as “***I don’t know if I should be telling you this*” or “*I know I shouldn't but*** *…*” frequently preceded discussion about workarounds. Fear of professional retribution and consequences was often linked to these discussions. To illustrate, having just observed a nurse complete a medication round:

16:40—I asked about the times when the nurses take the medication trolley to the bedside and sometimes that they don't. “**We are naughty**, you are supposed to take the trolley to the bedside and check the armband for every single medication even Panadol.” I pursue any reasons why sometimes it happens and others not … “it’s just some staff. In the morning I start and **I try to do the right thing**”. [(DC/FNO): 68 Evening Shift])

The tension nurses expressed about using workarounds was exemplified in nurses' accounts of the gap between what was taught in university as “ideal care” and what was realistic in everyday practice. Delivering care in the “real” world, nurses argued, required workarounds to get the job done, and it was evident that workarounds with the EMMS flowed into other areas of care delivery. The nurse repeatedly shook her head as she explained the following while sitting at the nurses' station having completed the evening medication round:

There is a gap between education during training about how to give medications and the reality of when you get on the wards. I would like to say, yes the 5Rs are important, but I would like to know which are the most important because you don't have time.

[(DC/FNO): 71_Evening Shift])

The complexity of the experience of tension when using workarounds was illustrated by a nurse participating in the observations, a focus group and an individual interview. The nurse showed mixed views about workarounds—saying in a one-on-one interview that they never used them, even though they were seen doing so, and agreeing in a group discussion with senior staff that workarounds are a normal part of daily work.In doing so they highlighted the gap between what nurses espoused publicly, the sacred, and the profane, what they talked about off the record and what they actually did (70).

Some nurses managed the tension between organisational expectations of timely medication administration (“*work-as-imagined*”) and the realities of clinical practice (“*work-as-done*”) by administering medications early but delaying documentation in the EMMS until the scheduled time. This workaround allowed them to appear compliant while maintaining workflow efficiency. To mitigate the risk of error, nurses often communicated informally with colleagues to confirm that medications had already been given. Although the EMMS recorded the time medications were signed off, EMMS implementation stakeholders acknowledged that these records did not always reflect real-time practice. This highlights a disconnect between system data and actual clinical activity:

I can, from a system point of view, have a look … From a system point of view, you can see what's occurred and then you have to make some inferences as to what the actual workflow behind that was. (Focus Group: EMMS implementation stakeholders_ID_2)

Nurses expressed frustration, feeling trapped in a “damned-if-you-do and damned-if-you-don’t” situation where they had to justify using workarounds in an under-resourced system. They believed workarounds were acceptable when used safely by experienced staff, but not in all situations. For example:

“To be honest, if we went two to the bedside for every single IV medication and infusion, the patients would die from not getting their medications … because there isn't enough time … You might both check and do it by the book but the patient would be dead because they wouldn't have got half of their meds…” [Nurse 69] told me that for some of the medications someone definitely goes with the administering nurse—ALWAYS because those medications are more complex or dangerous or they involve steps that need to be exactly … This increases the chance of compliance, but you must always be asking yourself … “what is better for the patient, what is safer for the patient? Constantly weighing it up”. [(DC/FNO): _69_Evening shift])

[Nurse 44] says “I know how it is meant to be done BUT we have to get care to our patients. We do the best we can we choose the best way to get things done. We can't do it all”. [(DC/FNO): _44_Evening shift])

#### Ambivalence

Some workarounds did not appear to evoke an emotional response or attitude. Several nurses described theirs and their colleagues' workarounds using “matter of fact” tones, without using qualifiers or words that depicted emotions. These descriptions of workarounds were not couched in positive or negative terms, rather they were part of the adaption to the EMMS, and to delivering care at a broader level, and were sometimes presented as a “fait accompli”.

Legibility, and it is quite user friendly now that we've ironed out all the bugs and worked out our shortcuts and ways around doing things and stuff like that. (Interview: Nurse_61)

While such statements may initially appear to reflect increased familiarity or efficiency with the system, closer examination reveals that the term “shortcuts” refers to informal, unsanctioned adjustments to the intended use of EMMS. These adaptations represent workarounds because they deviate from prescribed protocols and workflows.

While some expressed that they did not use workarounds themselves, they sounded noncommittal about their colleagues doing so. For example, Nurse_42 reported that whether nurses worked around the policy that required them to take the WOW to the bedside was not considered problematic, as long as they did not breach “the standards” by which they judge their performance as a good nurse,—that is, patient-centred, safe, team player and able to deliver care efficiently.

I've seen some people—they have their own way. As long as I—personally I don't care, if they're not with their medication, the electronic medication, or take the COW or whatever—how they want to do it, as long as they don't breach ‘the standards’. (Interview: Nurse_42)

When describing her colleagues' use of workarounds, Nurse_53 spoke in a neutral and non-judgemental tone, simply acknowledging that some nurses engaged in workarounds while others did not.

I don't personally, but people do their own practices. Sometimes you will see people writing, putting it in a kidney dish and writing a number or a patient's drug and maybe racing them up because they've got all their meds from the medication room or something or other. I mean it's rare. They might line them up like the Heparins or Calciparines or Clexanes and things ready to go with numbers and things. I don't do that. I just collect what I need and palm them out as I go. (Interview: Nurse_53)

#### Positive emotions

Many nurses expressed positive feelings and attitudes to workarounds, describing them in such terms as “resilient” and “resourceful”**.**

Nurses are resilient—they can ensure that the basic care is given and then write a note to sign off a medication later. (Interview: Nurse_26)

Personally, I don't know shortcuts through it but then again—or workarounds or whatever they call it … I don't doubt that nurses and medical staff being extremely resourceful people, that if they're there, they'll find them quite quickly. (Interview: Nurse_20)

For some, workarounds were viewed as a way of “thinking outside the box” (Field Notes: Observation_77_Evening shift) and their use indicative of an ability to problem-solve and personalise care through innovation. Nurse 50 drew on the notion that in a fluid working environment nurses use workarounds to adapt to and navigate the unexpected:

Nurses put their workarounds in place because they're dealing with any given situation that is never the same. (Interview: Nurse_50)

Having recourse to workarounds that involved more than one nurse was often perceived as a proxy for being a team player and indicated trust in each other's competency as a nurse. For example, Nurse 31 interpreted her colleagues' workaround of the policy requiring them to witness her administrating a medication as demonstration that they trusted her professional practice:

I see that my colleague trusts me … it's a kind of validation, like a respect—they trust you, as an RN. (Interview: Nurse_31)

## Discussion

This study examined how nurses feel towards workarounds when administering medication using EMMS in their everyday clinical practice. We know that when administering medication using EMMS nurses deploy two type of workarounds—primary and secondary—alone or in combination, to achieve multiple purposes (48). Primary workarounds come into play when circumventing a barrier to achieving a goal; and secondary workarounds are mobilised to overcome barriers produced by using a primary workaround (48). The reasoning nurses provide for applying workarounds is to be a “good nurse”, to be time-efficient, safe, patient-centred or to be a team player (1, 48).

This paper adds ethnographic texture to these findings by reporting how nurses feel about using workarounds to be a “good nurse”. It highlights that whether nurses had recourse to, or avoided using workarounds, there were simultaneous feelings of ambivalence, negativity, and positivity, depending on context, setting and type of workaround mobilised. The findings highlight how the complex and interconnected dynamics of individual actions, team priorities and orientations, and organisational constraints shape the practice environment—one that both enables the creation of workarounds and simultaneously tends to deny their existence.

An omnipresent feature of nursing practice is fear of professional retribution, particularly if an adverse event occurs (37, 41–43, 45–47). These incompatible realities leave nurses facing an invidious choice: to be a “good nurse”, sometimes bypassing rules to provide better care, potentially incurring consequences for not following policy; or to strictly follow the rules and cause potential care delays, incurring the frustration of their colleagues and potential patient safety issues. This situation can lead to internal stress, which is a potential contributor to burnout and professional disengagement, as we have documented here.

Our findings reveal that nurses had conflicting feelings about workarounds—both their own and their colleagues'. Most described a tension between the perceived necessity of workarounds, the hesitation and unwillingness to deviate from policy, and the desire to be a “good nurse”. On the one hand workarounds were seen to demonstrate an ability to “think outside the box” and were the hallmark of an expert: a mindful nurse who doesn't “blindly follow the rules”. On the other hand, as deviations from the rules, workarounds were also conceptualised as unsafe both for patients and nurses themselves and, therefore, unprofessional. These conflicting feelings can lead to anxiety.

In her seminal work examining the high levels of stress and anxiety among nurses, Menzies (71) described social defences that nurses employ to reduce anxiety associated with their work. These include eliminating decisions through ritual task performance; reducing the weight of responsibility in decision-making by checks and counterchecks; and purposeful obscurity in the formal distribution of responsibility (71). Thus, it appears that nurses have long relied on “following the rules”, particularly in relation to medication administration, to manage tension inherent in their work. Yet, as demonstrated in our study, nurses perceive that they need to work around rules to deliver care, to be a good nurse. It is unsurprising then that needing to work around the rules creates stress, and anxiety.

### Tension, burnout and disengagement

Workarounds have been described as the byproduct of workload pressures and as “survival mechanisms” (58) for healthcare professionals navigating a paucity of resources, inadequate systems and professional burnout. Tension arises in professionals caught between needing to use workarounds but in doing so breaching organisational rules. The study has noted the link between burnout and workarounds, established in the literature (43, 52, 53). The intricate dynamics and impact, for individuals and nursing cohort, needs further investigation.

### Potential solutions

On the basis of the foregoing, how might we formulate a strategic way forward?

**De-implementing low-value care/policies that do not support patient safety:** Focusing on and removing mandated nurses' work that is not essential or evidence based could help alleviate the pressure to use workarounds. For instance, recent research indicates that requiring double-checking of medication administration at the bedside offers minimal safety benefits if not done independently (72). The findings of our study highlighted the stress and professional vulnerability nurses experience when they felt forced to work around medication double checking policies because of a lack of resources and the need to administer medication in a timely way. These findings are supported by an international study conducted in Australia and the United Kingdom which identified double-checking medication as a safety practice that health care staff perceived to be of low value (sometimes because they could not complete it independently) (73). Simplifying procedures and removing low-value safety practices could reduce the need for nurses to use workarounds. However, stopping practices introduced to reduce uncertainty and that are perceived to increase patient safety (71) (even when not evidence based) is notoriously difficult (74). We need to specify the conditions under which the de-implementation of low-value practices can occur, and how doing so reduces nurses' need to use workarounds.

**Implementing the “Traffic Light Model”**: Adapting the “Traffic Light Model” used in antimicrobial prescribing (75) can shift us along the continuum of managing workarounds more effectively. This model categorises policies into: **Green Policies**: Allow workarounds if they are beneficial and counter secondary harms; **Orange Policies**: Permit cautious workarounds with senior guidance; **Red Policies**: Prohibit workarounds entirely. This approach acknowledges the inevitability of workarounds and provides a structured way to manage them, ensuring patient safety and nurse autonomy.

**Preparing Newly Graduated Nurses**: Given the high rate of attrition among newly graduated nurses due to disillusionment and dissatisfaction (76, 77), it is crucial to expose them to the reality of workarounds before they graduate. Creating realistic expectations and educating neophyte nurses about the challenges they will face and preparing them to navigate these challenges can reduce stress and improve retention rates (78). This preparation should include training on how to identify and manage workarounds safely and effectively.

**Enhancing psychological safety and autonomy** in the workplace is crucial for supporting nurses effectively. Encouraging open communication and providing robust support systems are essential strategies to help nurses manage stress and maintain their professional engagement. By fostering an environment where nurses feel safe to voice their concerns and have the autonomy to make decisions, we can contribute to the reduction in burnout and the need for workarounds (58). The results of our study might assist clinical leadership in acknowledging workarounds, understanding their underlying triggers, and working towards reconciling official procedures with real-world situations (79). This approach can help nurses in clinical practice reflect on and reconcile the demands of their organisations with their patient-oriented professional needs (59).

**Change the dichotomous conceptualisation of workarounds** to align with other research which underscores the complexity of workarounds in healthcare settings. Boonstra et al. (2021) (28) present a typology of enduring workarounds in Electronic Health Records (EHR), helping users and managers differentiate between harmful and less harmful workarounds to inform their decisions on discouraging or legitimising them. Additionally, Tucker et al. (2020) (36) suggest that workarounds used to navigate operational failures can lead to more positive patient outcomes, while those deployed to avoid processes are associated with negative outcomes. Investigating coping strategies could help managers and employees manage job stress and reduce burnout in the healthcare sector, thereby enhancing effectiveness and efficiency through safety workarounds (80).

Aligned with the findings of this study, Goff et al. (2021) (81), drawing on interview data from a policy pilot in general practice within the National Health Service in England, challenge the dichotomous conceptualisation of workarounds in relation to access to care. They argue that workarounds can both support and undermine policy pilots, making them inherently political as employees must balance the consequences for themselves and the wider organisation.

### Strengths and limitations

Most studies examine medication administration on day and evening shifts (27) on weekdays (40). This study was conducted across all shifts and days of the week enabling the capture of primary and secondary workarounds. The strength of *in situ* studies of behaviour is their capacity to understand context but this limits generalisability to other settings (82). To minimise this limitation, the study sample covered six units in two hospitals. Not every type of workaround was seen; rather it sampled for variation.

A potential limitation of this study was the Hawthorne effect, which has been observed in some observational studies examining nurses' compliance with interventions (70). However, prolonged engagement in the field allowed nurses time to become accustomed to the researcher's presence, thereby reducing the likelihood of sustained behaviour changes aimed at presenting a more favourable image (83).

Pre-planned observations may mean some aspects of care may have gone unnoticed; especially with a sole field data collector. To minimise these effects regular debriefing with the research team was conducted and a reflexive approach taken. The researcher may not have seen surreptitious behaviours that complied with policies but manifested as workarounds. In some circumstances the researcher was limited to visible observation data and was unable to hear communication between nurses and patients.

## Conclusions

Workarounds are an inevitable part of health care delivery. The rhetoric that workarounds are always harmful and unprofessional is not only unrealistic but indefensible and causes tension in nurses. This tension will likely contribute to stress and burnout, which leads to more workarounds and unsafe practices. Negative attitudes towards workarounds must be addressed, and a more open and nurse-centred discussion about their use encouraged.

## Data Availability

The datasets presented in this article are not readily available because the authors do not have ethical approval to share the qualitative data sets. Requests to access the datasets should be directed to deborah.debono@uts.edu.au.
